# Optimization of Ultrasonic-Assisted Extraction of Crude Flavonoids from Moutai Distillers Grains and Evaluation of Their Antioxidant and Anti-Inflammatory Activities

**DOI:** 10.3390/foods14132316

**Published:** 2025-06-30

**Authors:** Ju Guo, Wei Liu, Hehe Li, Qinfei Ke, Feng Chen, Qingran Meng, Xingran Kou

**Affiliations:** 1 School of Brewing Engineering, Moutai Institute, Huairen 564507, China; guoju@mtxy.edu.cn (J.G.); 13466010734@163.com (W.L.); 2 School of Food and Health, Beijing Technology & Business University, Beijing 100048, China; lihehe@btbu.edu.cn; 3 Department of Food, Nutrition, and Packaging Sciences, Clemson University, Clemson, SC 29634, USA; kqf@sit.edu.cn; 4 Collaborative Innovation Center of Fragrance Flavour and Cosmetics, School of Perfume and Aroma Technology, Shanghai Institute of Technology, Shanghai 201418, China; fchen@clemsn.edu

**Keywords:** Moutai distillers grains, crude flavonoid extract, extraction optimization, in vitro antioxidant, in vitro anti-inflammatory

## Abstract

Distillers grains are the main by-products of the brewing industry, with a large output but a low degree of resource utilization. Exploring more efficient comprehensive utilization technologies for distillers grains is of great significance for increasing the added value of the brewing industry. This study took Moutai distillers grains as the research object, and the ultrasonic-assisted extraction process of crude flavonoids from distillers grains was first optimized. On this basis, the in vitro antioxidant and anti-inflammatory effects of the crude flavonoid extract were further explored. The results show that the optimal process parameters for ultrasonic-assisted extraction of crude flavonoids were an ethanol concentration of 95%, liquid-to-solid ratio of 26 mL/g, and ultrasonic time of 36 min (with a fixed ultrasonic power of 500 W). Under such conditions, the yield of crude flavonoid extract from Moutai distillers grains was 25.39% ± 5.05%. In vitro antioxidant results showed that 2 mg/mL of crude flavonoid extract had good DPPH and ABTS free radical scavenging activities (78.17% and 75.21%, respectively). In vitro anti-inflammatory results showed that 0.5% crude flavonoid extract (the survival rate of HaCaT cells treated with this concentration was greater than 80%) significantly reduced the secretion of the inflammatory factors IL-6 and IL-1β induced by TNF-α and IFN-γ. In summary, this study showed that Moutai distillers grains may provide easily accessible and inexpensive raw materials for the functional food and cosmetic industries.

## 1. Introduction

As one of the six major distilled spirits in the world, Chinese Baijiu is usually made from wheat, sorghum, rice, and other raw materials through cooking, saccharification, fermentation, distillation, aging, storage, and blending [1]. The solid residue formed after the brewing of Chinese Baijiu is distillers grains [2]. In recent years, with the increase in the output of Chinese Baijiu, the output of distillers grains have also increased sharply. According to statistics, the annual output of Chinese baijiu exceeds 8.54 billion L, and the resulting distillers grains are about 25.61 million tons [3]. As the largest by-product of the Chinese Baijiu industry, distillers grains contain a large amount of crude fiber, crude protein, crude fat, and other nutrients due to incomplete fermentation [4]; at the same time, distillers grains usually have high water content and high acidity and are prone to corruption and mildew [5]. If not properly handled in time, it will not only easily cause resource waste, but also may cause serious environmental pollution and other problems [6].

Traditionally, distillers grains are mainly used directly as feed or fertilizer, but this method has low added value and a low utilization rate [7]. In recent years, some novel technologies have gradually begun to be applied to the comprehensive development of distillers grains in order to maximize the utilization of resources. Studies have shown that after a certain treatment, distillers grains can be used to produce silage and bacterial protein feed. This makes full use of the nutritional value of distillers grains, effectively reduce breeding costs and improve breeding benefits [8]. The large amount of lignin and cellulose in distillers grains has good adsorption properties, and the adsorbent materials produced can effectively remove heavy metals from water and soil, playing a good role in environmental protection [9]. In addition, distillers grains can also be used as a renewable energy source for the production of biogas [10], ethanol fuel [11], and new energy battery electrode materials [12]. These innovative methods have provided new ideas for the comprehensive development of distillers grains to a certain extent, but at the same time, there may also be problems, such as high processing costs, large investments, and slow returns. Therefore, further exploring more efficient and comprehensive utilization technologies for distillers grains is very important for increasing the added value of the Chinese Baijiu industry.

Among the many types of Chinese Baijiu, Moutai Town in Guizhou Province is a world-famous production area of Chinese Baijiu. In addition to its unique environmental factors that are conducive to the habitat and reproduction of brewing microorganisms, it also benefits from its unique brewing raw materials, namely glutinous sorghum [13]. The high concentration of natural active ingredients contained in it not only gives the Chinese Baijiu a unique aroma, but also makes the formed distillers grains rich in natural active ingredients such as polyphenols and flavonoids [14]. Ni et al. (2024) studied the changes in natural products such as polyphenols and flavonoids in the fermentation substrate during the processing of Moutai, and a total of five phenolic acids, including ferulic acid and caffeic acid, four flavonoids, including luteolin and apigenin, and three proanthocyanidins, including apigeninidin, were identified [15]. Chen et al. (2023) identified rutin, quercetin, naringenin, and dihydroquercetin from the distillers grain polyphenol extract of Moutai using liquid chromatography–high-resolution electrospray ionization mass spectrometry technology [16]. Numerous studies have demonstrated that natural products, particularly plant-derived polyphenols and flavonoids, possess potent biological activities, including antioxidant, antibacterial, hepatoprotective, antihypertensive, and hypoglycemic effects [17,18]. These bioactive properties have promoted in the search for health-promoting compounds [19], offering promising avenues for the high-value utilization of distillers grains. It is worth noting that compared with extracting and preparing natural products such as polyphenols and flavonoids from traditional plants, the polyphenols and flavonoids in distillers grains mainly come from brewing grains and microbial transformation, with relatively stable composition and content. This is conducive to the stable acquisition and further application of high-quality natural products. Therefore, the development of active natural products in distillers grains not only overcomes the shortcomings of traditional plant extraction, such as high raw material costs and seasonal restrictions, but may also increase the added value of Chinese Baijiu and other distillers grains, and to a certain extent, solve the problems of resource waste and environmental pollution caused by distillers grains.

In this study, the distillers grains of Moutai were used as raw materials, and the extraction of crude flavonoids was optimized using ultrasound-assisted extraction technology. Then, liquid chromatography–mass spectrometry (LC-MS) was used to analyze the types of flavonoids in the crude extract. Third, the antioxidant effects of the crude flavonoid extract were evaluated using chemical and cytological methods. Finally, HaCAT cells were used to evaluate the anti-inflammatory effect of the crude flavonoid extract. The study aimed to provide a theoretical basis for the high-value utilization and in-depth development of distillers grains.

## 2. Materials and Methods

### 2.1. Materials

Moutai distillers grains (the main brewing grains were wheat and waxy sorghum) were supplied by a certain distillery in Moutai Town, Guizhou Province, China. The distillers grains were dried at 60 °C until the moisture content was less than 10%. The dried samples were pulverized and passed through a 60-mesh sieve for later use.

Vitamin C, 1,1-diphenyl-2-picrylhydrazyl (DPPH) and 2,2′-azinobis-(3-ethylbenzthiazoline-6-sulphonate) (ABTS) were purchased from Meryer (Shanghai) Chemical Technology Co., Ltd. (Shanghai, China). The human immortalized keratinocyte HaCaT cell line was purchased from the Shanghai Institute of Biochemistry and Cell Biology (Shanghai, China). Dulbecco’s modified eagle medium (DMEM), fetal bovine serum, and phosphate buffer solution were purchased from Shanghai Titan Scientific Co., Ltd. (Shanghai, China). TNF-α, IFN-γ, and CTS™ GlutaMAX™ Supplement was purchased from Thermo Fisher Scientific Inc., Shanghai, China. Cell counting kit-8 (CCK-8), interleukin-6 (IL-6) kit, and interleukin-1β (IL-1β) kit were purchased from Shanghai Enzyme-linked Biotechnology Co., Ltd. (Shanghai, China). Other reagents were of analytical grade.

### 2.2. Single-Factor Experiment

Aliquots of crushed distillers grains were weighed and mixed with a certain volume fraction of ethanol solution in a certain liquid-to-solid ratio (mL/g). The mixture was subjected to ultrasound-assisted extraction (UC-22.5H, 500 W, Shanghai Titan Scientific Co., Ltd., Shanghai, China) for a certain period of time, and then centrifuged at 4000 r/min for 10 min (H2050R, Hunan Xiangyi Laboratory Instrument Development Co., Ltd., Changsha, China). The supernatant was then filtered and concentrated to dryness under reduced pressure (RE-52AA, Shanghai Yarong Biochemical Instrument Factory, Shanghai, China) to obtain a crude flavonoid extract from distillers grains.

Taking the extraction yield of crude flavonoid extract from distillers grains as an indicator, the effects of the ethanol concentration (%), liquid-to-solid ratio (mL/g), ultrasonic time (min), and extraction runs on the extraction output were investigated. The ethanol concentration was set at 60%, 70%, 80%, 90%, and 100%; the solid-to-liquid ratio was set at 1:15, 1:20, 1:25, 1:30, and 1:35 (g:mL); the ultrasonic time was set at 20, 30, 40, 50, and 60 min; and the extraction runs were 1, 2, 3, 4, and 5.

### 2.3. Response Surface Optimization

According to the results of single-factor test, three factors, namely, ethanol concentration (A), solid-to-liquid ratio (B), and ultrasonic time (C), were selected for Box–Behnken response surface design. The levels of each factor were as follows: ethanol concentrations of 80%, 90%, and 100%; solid-to-liquid ratios of 1:20, 1:25, and 1:30 (mg/mL); ultrasonic times of 30, 40, and 50 min. A total of 17 groups of experiments were designed, with the crude flavonoid extract yield as the response value.

Finally, the total flavonoid content of the crude flavonoid extract from Moutai distillers grains under the optimal extraction process was estimated using the NaNO_2_-Al(NO_3_)_3_-NaOH method [20]. Briefly, 0.1 mL of the crude flavonoid extract was diluted to 2 mL, and 0.25 mL of 5% NaNO_2_ solution was added. After standing for 5 min, 0.25 mL of 10% Al(NO_3_)_3_ solution was added, and the mixture was kept standing for 6 min. Then, 2 mL of 0.5% NaOH solution was added. After agitating, the mixture was held at room temperature for 15 min. The absorbance was measured at 510 nm using a UV-1800 UV–Vis spectrophotometer (Shimadzu (China) Co., Ltd., Shanghai, China). The result was expressed as the equivalent of rutin contained in each gram of raw material according to the rutin calibration curve (*y* = 0.8541*x* + 0.0752, where x represents the rutin content (mg), and *y* represents the corresponding absorbance).

### 2.4. In Vitro Antioxidant Assays

#### 2.4.1. Determination of DPPH Free Radical Scavenging Capacity

The DPPH· scavenging experiment was conducted according to the method proposed by Meng et al. (2017) [21] with some modifications. Briefly, the crude flavonoid extract from distillers grains was diluted with anhydrous ethanol to obtain working solutions with concentrations of 0.03125, 0.0625, 0.125, 0.25, 0.5, 1, and 2 mg/mL. One milliliter of freshly prepared DPPH in methanol (0.1 mmol/L) was mixed with 0.2 mL of each work solution and vitamin C (positive control) in water (0.03125–2 mg/mL), separately. After thoroughly mixing, all the mixtures were kept in the dark for 60 min, and the absorbance was measured at 517 nm. The DPPH· scavenging activity was calculated as follows:

(1) $$DPPH\;radical\;scavenging\;capacity\left(\%\right)=\frac{A_{0}-(A-A_{b})}{A_{0}}\times 100\%$$

where *A*_0_ is the absorbance of DPPH· solutions without crude flavonoid extract samples, *A* is the absorbance of the crude flavonoid extract samples mixed with DPPH· solutions, and *A*_b_ is the absorbance of crude flavonoid extract samples without DPPH· solutions.

#### 2.4.2. Determination of ABTS Free Radical Scavenging Capacity

The ABTS· scavenging experiment was carried out based on the method of Meng et al. (2021) [22] with minor modifications. Briefly, ABTS· was firstly prepared by mixing potassium persulfate solution (2.6 mmol/L) and ABTS solution (7 mmol/L) for 16 h in the dark. Before use, ABTS· was diluted with phosphate buffer saline (pH 7.0) to the absorbance of 0.70 ± 0.02 (734 nm). Then, 0.1 mL of crude flavonoid extract samples and vitamin C (positive control) at various concentrations (0.03125–2 mg/mL) were added to 1.9 mL of the diluted ABTS· solution. After thoroughly mixing, the mixture was kept in the dark for 5 min, and the absorbance was measured at 734 nm. The ABTS· was calculated as follows:

(2) $$ABTS\;radical\;scavenging\;capacity\left(\%\right)=\frac{A_{0}-(A-A_{b})}{A_{0}}\times 100\%$$

where *A*_0_ is the absorbance of ABTS· solutions without crude flavonoid extract samples, *A* is the absorbance of the crude flavonoid extract samples mixed with ABTS· solutions, and *A*_b_ is the absorbance of crude flavonoid extract samples without ABTS· solutions.

### 2.5. In Vitro Anti-Inflammatory Assays

#### 2.5.1. Cytotoxicity Analysis

The cytotoxicity analysis of crude flavonoid extract from Moutai distillers grains was carried out based on the method proposed by Kumar and Singh (2025) [23] with some modifications. Briefly, HaCaT cells in the logarithmic growth phase and in good condition were inoculated into 96-well plates at a rate of 1 × 10^4^ cells per well and cultured under 37 °C and 5% CO_2_ (Heracell Vios 160i CR, Thermo Fisher Scientific Inc., Shanghai, China) for 24 h. Then, the culture medium was aspirated, and the cell was rinsed with PBS buffer three times. DMEM containing 0 (control group), 0.1%, 0.2%, 0.3%, 0.4%, 0.5%, 0.6%, 0.7%, 0.8%, 0.9%, and 1% of crude flavonoid extract from Moutai distillers grains was added. After culturing for 24 h, the supernatant was discarded, and 100 μL of CCK-8 solution was added to each well. After incubation at 37 °C for 1 h, the absorbance was determined at a wavelength of 450 nm using an enzyme-linked immunosorbent assay reader (Agilent Synergy H1, Temecula, CA, USA). The cell viability was calculated as follows:

(3) $$Cell\;viability\left(\%\right)=\frac{A_{s}-A_{b}}{A_{c}-A_{b}}\times 100\%$$

where *A*_s_ is the absorbance of HaCaT cells treated with crude flavonoid extract samples and CCK-8 solution; *A*_b_ is the absorbance of the crude flavonoid extract samples mixed with CCK-8 solution, and *A*_c_ is the absorbance of HaCaT cells treated with CCK-8 solution.

#### 2.5.2. Determination of Anti-Inflammatory Efficacy

The anti-inflammatory experiment was carried out based on the method of Hu et al. (2025) [24] with minor modifications. Specifically, HaCaT cells in logarithmic growth phase and in good condition were seeded in 48-well plates at 1 × 10^4^ cells/well. After culturing for 24 h at 37 °C and 5% CO_2_, the old culture medium was removed, and the cells were washed three times with PBS buffer. HaCaT cells were then pretreated with crude flavonoid extract from Moutai distillers grains (0.1, 0.2, 0.3, 0.4, and 0.5% in DMEM medium) for 2 h, and then the mixture of TNF-α and IFN-γ (1:1, the final concentration of both in the culture medium was 10 ng/mL) was added to stimulate the cells. After culturing for 24 h, the cell culture plates were centrifuged at 1000 r/min for 20 min (Eppendorf Centrifuge 5804 R, Hamburg, Germany), and the supernatant was collected. Then, the contents of IL-6 and IL-1β in the supernatant were detected according to the instructions of the kits.

### 2.6. Statistical Analysis

All the experiments were conducted for at least three times, and data are represented as mean values ± standard deviation (SD). Design-Expert software (V8.0.6, Stat-Ease, Inc., Minneapolis, MN, USA) was used for Box–Behnken design. Analysis of variance (ANOVA) and Duncan’s multiple range tests were adopted for statistical analysis (*p* < 0.05). Figures were plotted using Origin 2021 software (OriginLab Corporation, Northampton, MA, USA).

## 3. Results and Discussions

### 3.1. Single-Factor Experimental Analysis

#### 3.1.1. Effect of Ethanol Concentration on Crude Flavonoid Extract Yield

**Figure 1:**
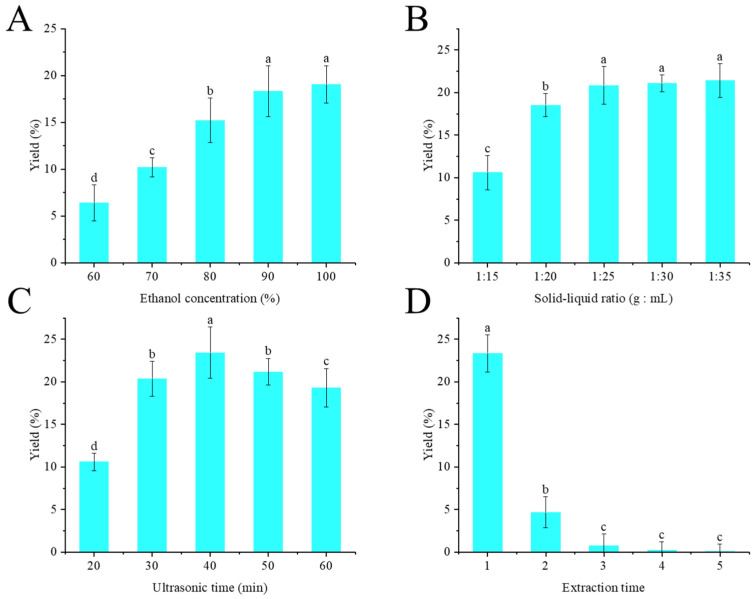
Effect of ethanol concentration (**A**), solid-to-liquid ratio (**B**), ultrasonic time (**C**), and extraction runs (**D**) on the extraction yield of crude flavonoid extract from distillers grains (Bars with the same letters indicate no significant difference (*p* > 0.05)).

The effect of ethanol concentration on crude flavonoid extract yield is shown in Figure 1A. Here, the solid-to-liquid ratio (g/mL) was set at 1:20, ultrasonic time was set at 30 min, and one extraction was performed. As the ethanol concentration increased, the extraction yield of crude flavonoid extract from Moutai distillers grains increased accordingly. When the ethanol concentration was 90%, the extraction yield of crude flavonoid extract from Moutai distillers grains reached the maximum value of 18.31% ± 3.72%. When the ethanol concentration was further increased, there was no difference in the yield of crude flavonoid extract from Moutai distillers grains (*p* > 0.05). Therefore, ethanol concentration of 90% was selected in this experiment.

#### 3.1.2. Effect of the Solid-to-Liquid Ratio on Crude Flavonoid Extract Yield

The effect of the solid-to-liquid ratio on crude flavonoid extract yield is shown in Figure 1B. Here, the ethanol concentration was set at 90%, ultrasonic time was set at 30 min, and one extraction was performed. The ratio of solvent to raw materials had a significant impact on the extraction efficiency. Within the range of 1:10–1:20 g/mL, with the increase of solvent dosage, the dissolution efficiency of the target component showed an upward trend, and the highest extraction yield of 20% ± 4.07% was achieved at 1:20. However, when the proportion of the solvent continued to increase, the extraction efficiency tends to be constant (*p* > 0.05). Therefore, a solid-to-liquid ratio of 1:20 was selected in this experiment.

#### 3.1.3. Effect of Ultrasonic Time on Crude Flavonoid Extract Yield

The effect of ultrasonic time on crude flavonoid extract yield is shown in Figure 1C. Here, the ethanol concentration was set at 90%, the solid-to-liquid ratio was set at 1:25 (g:mL), and one extraction was performed. Within the range of 20–40 min, with the increase in ultrasonic treatment time, the dissolution efficiency of crude flavonoid extract continued to rise and reached the maximum value of 23.73% at 40 min. Subsequently, the yield of crude flavonoid extract gradually decreased over time. The mechanical effect and cavitation of ultrasonic waves can promote the rupture of cell walls, thereby enhancing the dissolution efficiency of the target components. However, excessive ultrasonic treatment may also lead to the degradation of flavonoids [25]. Therefore, ultrasonic time of 40 min was selected in this experiment.

#### 3.1.4. Effect of Extraction Runs on Crude Flavonoid Extract Yield

The effect of extraction runs on crude flavonoid extract yield is shown in Figure 1D. Here, the ethanol concentration was set at 90%, the solid-to-liquid ratio was set at 1:25 (g:mL), and ultrasonic time was set at 40 min. Obviously, with the continuous increase in extraction runs, the yield of crude flavonoid extract from Moutai distillers grains gradually decreased. When the extraction exceeded three runs, the extraction yield hardly changed. From the perspectives of energy conservation, emission reduction, and sustainable development, only two extractions were carried out in the following experiments.

### 3.2. Box–Behnken Design for Optimization of Extraction Parameters

#### 3.2.1. Establishment and Analysis of the Response Surface Regression Model

The data were fitted by multiple regression using Design Expert 13 (Table 1), and the regression equation between the extraction rate of crude flavonoid extract from Moutai distillers grains and the investigated single-factor variable was established as follows: *y* = 24.20 − 1.74*x*_1_^2^ − 2.01*x*_2_^2^ − 3.34*x*_3_^2^ + 0.62*x*_1_*x*_1_ − 0.23*x*_1_*x*_2_ − 0.61*x*_2_*x*_3_ + 1.63*x*_1_ + 0.81*x*_2_ − 1.95*x*_3_.

The model *F*-value of 74.67 (Table 2) implied the model was significant, and there was only a 0.01% chance that an *F*-value this large could occur due to noise. *p*-values less than 0.0500 indicated that the model terms were significant. In this case, the order of influence of each factor on the extraction rate of crude flavonoid extract from Moutai distillers grains was as follows: ultrasonic time (*X*_3_) > ethanol concentration (*X*_1_) > solid-to-liquid ratio (*X*_2_); *X*_1_, *X*_2_, *X*_3_, *X*_1_*X*_2_, *X*_2_*X*_3_, *X*_1_^2^, *X*_2_^2^, and *X*_3_^2^ were significant model terms. The lack of fit *F*-value of 10.47 implied that the lack of fit was significant, and there was only a 2.30% chance that a lack of fit *F*-value this large could occur due to noise. The model’s *R*^2^ = 0.9897, indicating that the actual value of the extraction rate of crude flavonoid extract from Moutai distillers grains had a good fit with the predicted value. The predicted *R*^2^ of 0.8519 was in reasonable agreement with the adjusted *R*^2^ of 0.9764, i.e., the difference was less than 0.2. Adeq Precision measures the signal to noise ratio, and a ratio greater than 4 is desirable. In this case, an Adeq Precision value of 23.669 indicated an adequate signal. Hence, this model can be used to navigate the design space.

#### 3.2.2. The Influence of Factor Interaction on the Extraction Yield

**Figure 2:**
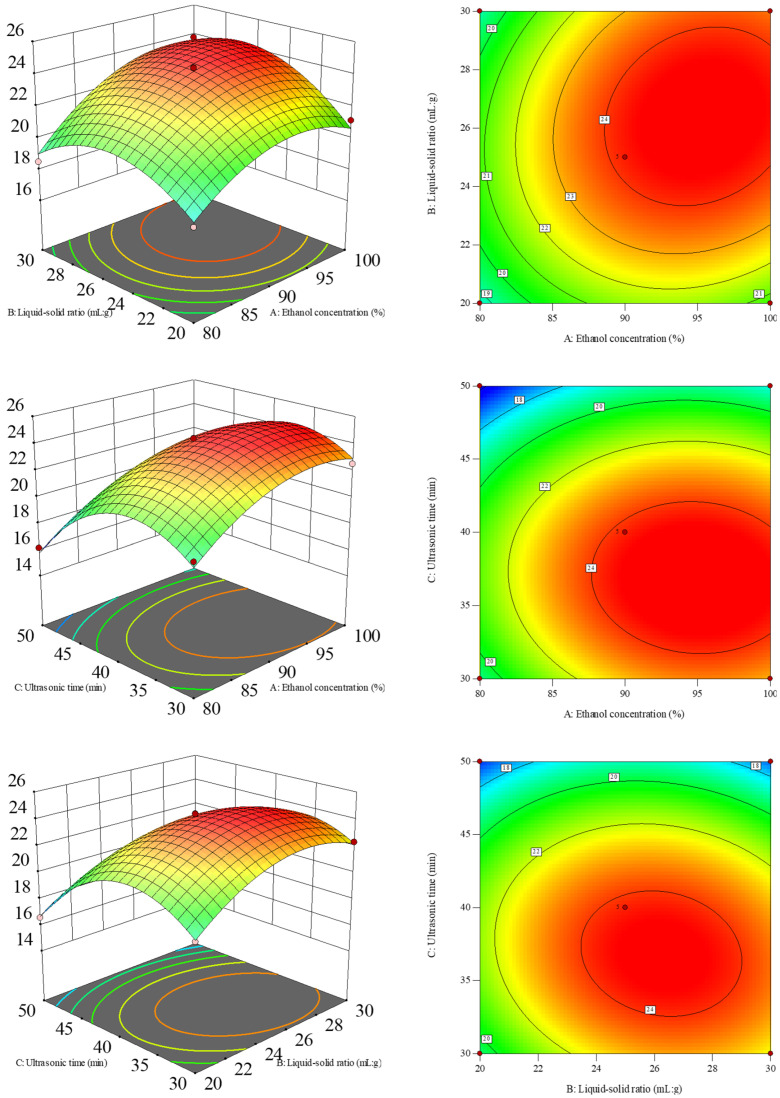
Response surface plots and contour plots of the crude flavonoid extract from Moutai distillers grains affected by ethanol concentration (*X*_1_), liquid-to-solid ratio (*X*_2_), and ultrasonic time (*X*_3_).

The steeper the response surface map is or the more elliptical the contour map is, the more significant the interaction is. As can be seen from Figure 2, the results of the response surface analysis showed that the interaction surfaces between the ethanol concentration and the liquid-to-solid ratio as well as the liquid-to-solid ratio and ultrasonic time had significant steepness characteristics. The contour lines presented an obvious elliptical distribution pattern, indicating a significant interaction relationship between the two (*p* < 0.05). In contrast, the response surface of the ethanol concentration and ultrasonic time showed a gentle changing trend. The contour line shape was approximately circular. This feature indicated that the interaction between the two has not reached a significant level (*p* > 0.05). This result was consistent with the analysis conclusion presented in Table 2.

#### 3.2.3. Verification Test

According to the analysis of the Box–Behnken model, the optimal extraction process parameters for the extraction yield of crude flavonoid extract were ethanol concentration of 95.5%, a liquid-to-solid ratio of 26.69 mL/g, and ultrasonic time of 36.58 min. Under these parameter conditions, the extraction yield of crude flavonoid extract from Moutai distillers grains was 25.12%. For the convenience of practical feasibility, the above parameters were corrected to an ethanol concentration of 95%, a liquid-to-solid ratio of 26 mL/g, and an ultrasonic time of 36 min. Three verification tests were conducted by modifying the process parameters, and the extraction rate of crude flavonoid extract from Moutai distillers grains was 25.39% ± 5.05%, with a relative error of 1.07% compared to the theoretical value, indicating that this process was stable and feasible. Under such optimal conditions, the total flavonoid content in the crude flavonoid extract from distillers grains was 256.8 ± 13.31 mg/g raw material (rutin equivalent).

### 3.3. In Vitro Antioxidant Activities

#### 3.3.1. DPPH Radical Scavenging Activity

**Figure 3:**
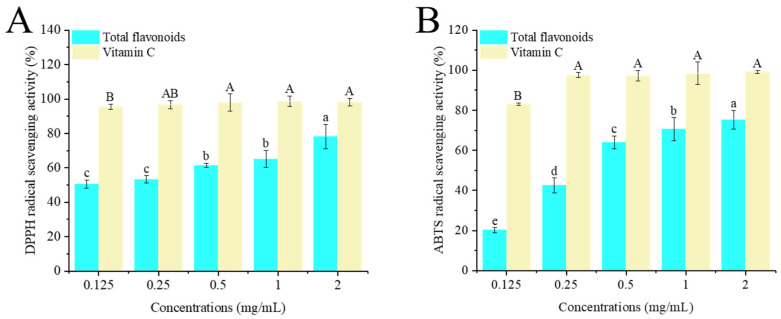
Antioxidant activities of the crude flavonoid extract from Moutai distillers grains. (**A**) DPPH free radical scavenging activity, (**B**) ABTS free radical scavenging activity (Bars with the same letters indicate no significant difference (*p* > 0.05)).

The principle of the DPPH assay refers to the evaluation of the scavenging capacity of antioxidant substances by detecting the change in absorbance of stable DPPH free radicals after their reaction with target substances. When reacting with target substances, DPPH radicals will be cleared, the absorbance will decrease, and the degree of reaction is positively correlated with the antioxidant capacity [26]. As shown in Figure 3A, within the concentration range of 0.125–2 mg/mL, the scavenging ability of crude flavonoid extract from Moutai distillers grains on DPPH radicals increased with the increase in concentration, showing a good dose–response relationship. However, it was lower than the scavenging rate of vitamin C on DPPH radicals at the same concentration. The scavenging rate of 2 mg/mL crude flavonoid extract from Moutai distillers grains on DPPH free radicals was 78.17%, indicating that the crude flavonoid extract from Moutai distillers grains had a good scavenging effect on DPPH free radicals.

#### 3.3.2. ABTS Radical Scavenging Activity

The antioxidant principle of ABTS refers to the fact that under the action of oxidants, ABTS will generate a blue–green free radical ABTS·^+^. The presence of antioxidant substances can inhibit the formation of ABTS·^+^, thereby reducing the absorbance of ABTS·^+^. By measuring the absorbance change in ABTS·^+^, the total antioxidant capacity of the sample can be evaluated and compared with an antioxidant standard to obtain the relative value of the sample’s antioxidant capacity [27]. In this study, within the concentration range of 0.125–2 mg/mL, the scavenging ability of the crude flavonoid extract from Moutai distillers grains on ABTS radicals increased with the increase in concentration, showing a good dose–response relationship (Figure 3B). The scavenging rate of 2 mg/mL crude flavonoid extract from Moutai distillers grains on ABTS free radicals was 75.21%, indicating that crude flavonoid extract from Moutai distillers grains had a good scavenging effect on ABTS free radicals.

### 3.4. The Effects of Crude Flavonoid Extract on the Viability of HaCaT Cells

**Figure 4:**
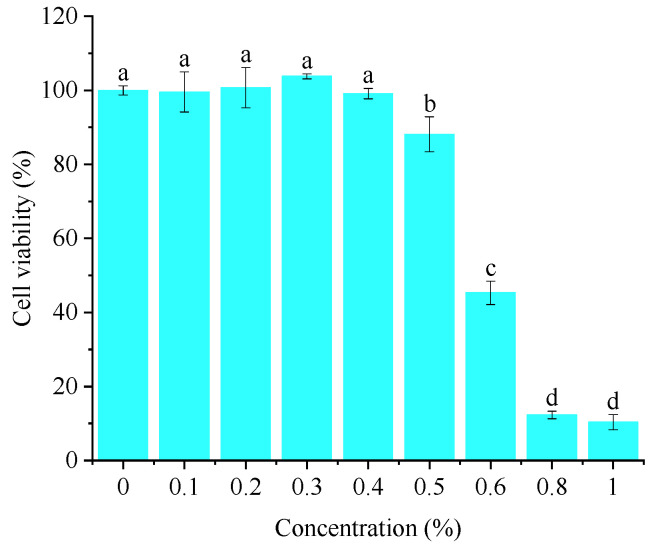
Effect of crude flavonoid extract on the viability of HaCaT cells (Bars with the same letters indicate no significant difference (*p* > 0.05)).

As can be seen from Figure 4, the inhibition rate of crude flavonoid extract from Moutai distillers grains on HaCaT cells gradually increased with the increase in concentration. When the concentration was greater than 1%, the cell survival rate was less than 15%; when the concentration was 0.5%, the HaCaT cell survival rate was >80%. It can be considered that crude flavonoid extract from Moutai distillers grains had little effect on the activity of HaCaT cells within the concentration range of ≤0.5%. Therefore, a concentration of 0.1–0.5% was selected for subsequent anti-inflammatory effect evaluation.

### 3.5. In Vitro Anti-Inflammatory Analysis

**Figure 5:**
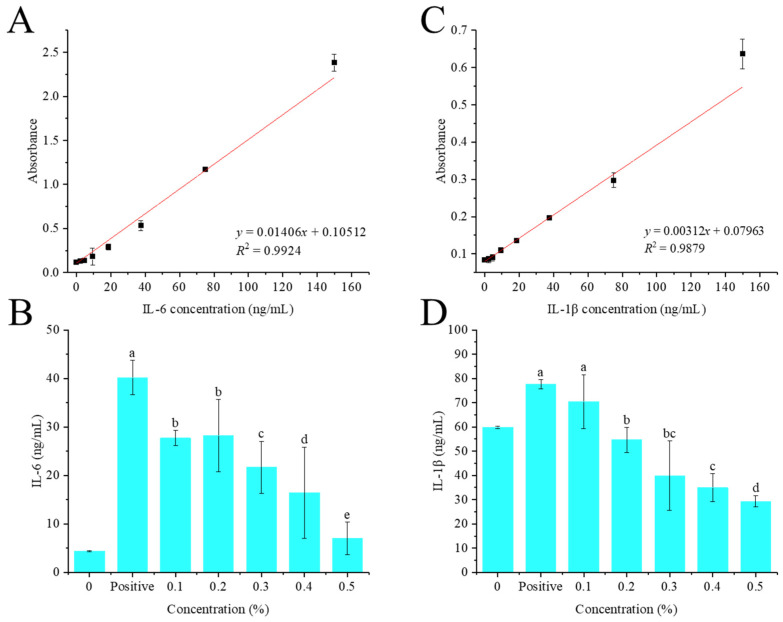
Anti-inflammatory activities of the crude flavonoid extract from Moutai distillers grains. (**A**,**C**) Standard curves of IL-6 and IL-1β; (**B**) the inhibitory effect on IL-6; (**D**) the inhibitory effect on IL-1β (Bars with the same letters indicate no significant difference (*p* > 0.05)).

As shown in Figure 5B, the concentration of IL-6 produced by normal HaCaT cells was only 4.42 ng/mL. TNF-α and IFN-γ treatment can significantly (*p* < 0.01) induce HaCaT cells to secrete IL-6, up to 40.15 ng/mL, which was 9.08 times that of the blank control group. Compared with the positive control group, 0.1–0.5% of crude flavonoid extract from Moutai distillers grains can significantly reduce the production of IL-6 in HaCaT cells by 31.04%, 29.77%, 45.05%, 59.00% and 82.56%, respectively (*p* < 0.05), and a dose-dependent effect was observed, indicating that crude flavonoid extract from Moutai distillers grains can effectively inhibit the production of IL-6 in HaCaT cells induced by TNF-α and IFN-γ.

Similarly, compared with the positive control group, 0.1–0.5% of crude flavonoid extract from Moutai distillers grains can significantly reduce the production of IL-1β in HaCaT cells by 9.28% (*p* > 0.05), 29.59% (*p* < 0.05), 48.60% (*p* < 0.05), 55.04% (*p* < 0.05), and 62.24% (*p* < 0.05), respectively, and showed a dose-dependent effect (Figure 5D). These results indicated that crude flavonoid extract from Moutai distillers grains can effectively inhibit the production of IL-1β in HaCaT cells induced by TNF-α and IFN-γ.

## 4. Discussion

Flavonoids are widely found in plants and have multiple pharmacological effects, such as improving blood circulation, lowering cholesterol, anti-atherosclerosis effects, delaying aging, and anti-oxidation effects [28], and are widely used in medicine, health foods, cosmetics, and other fields [29]. Many medicinal and edible plants (such as ginkgo [30], etc.) have long been verified to have biological activities of flavonoid components, which are in line with traditional Chinese medicine theory and modern health concepts. However, the growth of plants is significantly affected by the change of seasons, and they are only suitable for harvesting in certain seasons [31]. Harvesting too early or too late will affect the content and activity of flavonoids in plants. Environmental factors (light, soil, precipitation, altitude, temperature difference between day and night, etc.) are also critical to plants. At the same time, environmental pollution (such as industrial emissions and pesticide residues) will also interfere with plant metabolism, affect the synthesis and accumulation of flavonoids, and even introduce harmful substances, reducing the safety of extracts [32]. In addition, extreme weather events, such as heavy rain, drought, and cold waves, may lead to reduced or even total crop failure, seriously affecting the supply of raw materials for flavonoids. Therefore, developing new plant flavonoid resources is the core driving force to break through traditional bottlenecks and promote the sustainable development of the plant flavonoid industry. In this study, the ultrasonic-assisted extraction process of crude flavonoids from Moutai distillers grains was optimized based on the Box–Behnken design for the first time, and the results showed that the extraction rate of crude flavonoid extract from Moutai distillers was as high as 25.39% ± 5.05%. Under such optimal conditions, the total flavonoid content in the crude flavonoid extract from distillers grains was 256.8 ± 13.31 mg/g raw material (rutin equivalent), which was significantly higher than the yield of the crude flavonoid extract from general plants.

Zeng et al. (2024) [33] used ultrasonic-assisted extraction technology to study the extraction process of total flavonoids from *Radix puerariae* (the main active ingredient in *Radix puerariae*). Under the optimal conditions (ethanol concentration of 59%, liquid-to-solid ratio of 40 mL/g, ultrasonic time of 43 min (240 W)), the yield of total flavonoids from *Radix puerariae* was only 20.82 mg/g. Similarly, Xiang et al. (2024) [34] optimized the ultrasonic-assisted extraction conditions of total flavonoids from *Daphne genkwa* and found that the maximum total flavonoid content was 5.41 mg/g (rutin equivalent) under the optimal conditions involving an ultrasonic power of 225 W, extraction time of 30 min, liquid-to-solid ratio of 30 mL/g, extraction temperature of 60 °C, and ethanol concentration of 70%. Wen et al. (2023) [35] found that the total flavonoid content from *Ligusticum chuanxiong* Hort. was 3.07 mg/g under the optimal conditions of an ethanol concentration of 72%, extraction time of 61 min, liquid-to-solid ratio of 28 mL/g, extraction temperature of 40 °C, and ultrasonic power of 320 W. The total flavonoid content in the above plants and many other traditional medicinal plants is much lower than that in Moutai distillers grains, indicating that Chinese Baijiu distillers grains, specifically Moutai distillers grains, may become an important source of plant flavonoids. Traditionally, the raw materials for brewing Chinese Baijiu mainly include sorghum, wheat, peas, sweet potatoes, etc. [36], which are rich in flavonoid compounds. In addition, the flavonoid precursor substances existing in the above-mentioned raw materials can also be enzymatically hydrolyzed or metabolized into flavonoids by the microorganisms involved in the fermentation of Chinese Baijiu [15]. Therefore, the raw materials themselves and the metabolism of microorganisms may jointly lead to the high content of flavonoids in the Moutai distillers grains.

Additionally, the above unique biotransformation not only improves the problem of the overly single flavonoid structure in traditional plants, but also can significantly improve the biological activities of common plant flavonoids. In this study, crude flavonoid extract from Moutai distillers grains showed certain in vitro antioxidant and anti-inflammatory effects. Briefly, in vitro antioxidant results showed that 2 mg/mL of crude flavonoid extract had good DPPH and ABTS free radical scavenging activities (78.17% and 75.21%, respectively). In vitro anti-inflammatory results showed that 0.5% crude flavonoid extract could significantly reduce the secretion of the inflammatory factors IL-6 and IL-1β induced by TNF-α and IFN-γ. Researchers have also obtained total flavonoids with similar antioxidant and anti-inflammatory effects from plants. Wang et al. (2023) extracted the total flavonoids from *Artemisia argyi* stem and evaluated their antioxidant and anti-inflammatory activities. The results showed that the scavenging rate of 0.4 mg/mL total flavonoids from *Artemisia argyi* on DPPH free radicals was 95.13%, and the scavenging rate of 0.8 mg/mL total flavonoids from *Artemisia argyi* on ABTS free radicals was 88.39%. In terms of anti-inflammatory effects, 400 μg/mL of *Artemisia argyi* total flavonoids could significantly reduce the levels of NO, TNF-α, IL-1β, and IL-6 in RAW 264.7 macrophages stimulated by lipopolysaccharide [37]. In addition, *Gymnosporia montana* leaf and fruit extracts also showed similar antioxidant and anti-inflammatory activities [38]. Xu et al. (2022) found that propolis flavonoids exerted antioxidant and anti-inflammatory effects through the Nrf2 and NF-κB pathways [39].

In summary, the development of flavonoids from Chinese Baijiu distillers grains can break through the inherent mode of traditional flavonoids relying on plant cultivation and transform the ‘negative product’ of the brewing industry into a ‘positive asset’ in the field of natural active products. This innovation not only solves the problem of the sustainability of raw material supply, but also fits the development direction of the circular economy and green chemistry and can provide a new engine for the green transformation and functional upgrading of the traditional brewing industry. Although crude flavonoid compounds extracted from Moutai distillers grains can serve as a potential effective ingredient to attenuate and reduce free radicals and inflammatory conditions, their specific chemical composition and potential antioxidant and anti-inflammatory mechanisms are still unclear.

## 5. Conclusions

In this study, the optimal extraction parameters for a crude flavonoid extract yield of 25.39% ± 5.05% were an ethanol concentration of 95%, liquid-to-solid ratio of 26 mL/g, and ultrasonic time of 36 min with a fixed ultrasonic power of 500 W. In vitro antioxidant results indicated that crude flavonoid extract had good DPPH and ABTS free radical scavenging activities. In vitro anti-inflammatory experiments showed that the crude flavonoid extract could significantly reduce the secretion of the inflammatory factors IL-6 and IL-1β induced by TNF-α and IFN-γ. In summary, this study showed that Moutai distillers grains may provide easily accessible and inexpensive raw materials for the development of natural flavonoids in functional foods, cosmetics, and even drugs, and future research should focus on flavonoid identification and elucidate the mechanisms of the anti-inflammatory effects and other biological activities.

## Figures and Tables

**Table 1:** Experimental design and the yields of crude flavonoid extract from Moutai distillers grains.

Run Order	Variable Levels			Extraction Yield (%)	
	Ethanol Concentration (*X*_1_, %)	Liquid-to-Solid Ratio (*X*_2_, mL:g)	Ultrasonic Time (*X*_3_, min)	Actual Value	Predicted Value
1	90	20	50	16.59	16.70
2	90	25	40	24.41	24.20
3	90	20	30	19.28	19.38
4	80	30	40	18.51	19.01
5	80	25	30	19.61	19.22
6	80	20	40	18.35	18.64
7	90	25	40	24.15	24.20
8	90	25	40	23.91	24.20
9	90	30	30	22.33	22.22
10	80	25	50	16.17	15.77
11	90	30	50	17.19	17.09
12	100	30	40	23.79	23.50
13	100	25	30	22.53	22.93
14	90	25	40	24.14	24.20
15	100	25	50	18.17	18.56
16	100	20	40	21.15	20.65
17	90	25	40	24.39	24.20

**Table 2:** ANOVA results for the response surface quadratic model.

Source	Sum of Squares	d*f*	Mean Square	*F*-Value	*p*-Value
Model	144.87	9	16.1	74.67	<0.0001
*X*_1_-Ethanol concentration	21.13	1	21.13	97.99	<0.0001
*X*_2_-Liquid-to-solid ratio	5.2	1	5.2	24.12	0.0017
*X*_3_-Ultrasonic time	30.54	1	30.54	141.65	<0.0001
*X*_1_ *X*_2_	1.54	1	1.54	7.13	0.032
*X*_1_ *X*_3_	0.2116	1	0.2116	0.9816	0.3548
*X*_2_ *X*_3_	1.5	1	1.5	6.96	0.0335
*X*_1_^2^	12.73	1	12.73	59.05	0.0001
*X*_2_^2^	17.03	1	17.03	79.01	<0.0001
*X*_1_^2^	47.01	1	47.01	218.05	<0.0001
Residual	1.51	7	0.2156		
Lack of Fit	1.34	3	0.4462	10.47	0.023
Pure Error	0.1704	4	0.0426		
Cor Total	146.38	16			
Std. Dev.	0.4643		*R* ^2^	0.9897	
Mean	20.86		Adjusted *R*^2^	0.9764	
C.V. %	2.23		Predicated *R*^2^	0.8519	

## Data Availability

The original contributions presented in this study are included in the article. Further inquiries can be directed to the corresponding authors.
